# Efficacy of Esmolol Versus Nitroglycerin in Maintaining the Quality of Bloodless Surgical Fields in Middle Ear Surgery: A Randomized Controlled Trial

**DOI:** 10.7759/cureus.109959

**Published:** 2026-05-31

**Authors:** Mithra K, Lingaraj Sahu, Anjali Kumari, Laba K Nayak, Mousumi Das

**Affiliations:** 1 Anesthesiology, Kalinga Institute of Medical Sciences, Kalinga Institute of Industrial Technology (KIIT) University (Deemed to be University), Bhubaneswar, IND

**Keywords:** bloodless surgical field, controlled hypotension, esmolol, fromme-boezaart scale, heart rate control, middle ear surgery, nitroglycerin, tympanoplasty

## Abstract

Background

Middle ear surgeries such as tympanoplasty and mastoidectomy demand a bloodless operative field because even minimal bleeding under high-magnification microscopy can significantly impair visualization, prolong operative time, and increase the risk of iatrogenic injury. Controlled hypotension is widely accepted for minimizing bleeding; however, the optimal pharmacological agent in otological surgery remains debated. Esmolol, a cardio-selective beta-1 blocker, reduces heart rate (HR) and cardiac output without vasodilation, whereas nitroglycerin (NTG) frequently induces reflex tachycardia that may paradoxically increase microvascular bleeding.

Objective

To compare the efficacy of intravenous esmolol versus nitroglycerin in achieving and maintaining a bloodless surgical field in middle ear surgery under general anesthesia, with primary evaluation of Surgical Site Comfort (SSC) score, surgical and microscope duration, and secondary evaluation of intraoperative HR, mean arterial pressure (MAP), pre and post op hemodynamic variables.

Methods

A prospective, randomized, double-blind, parallel-group controlled trial was conducted at Kalinga Institute of Medical Sciences, Bhubaneswar. One hundred ASA I-II adult patients undergoing elective middle ear surgery were randomized to esmolol (Group A, n=50) or nitroglycerin (Group B, n=50) targeting MAP 60-65 mmHg. The surgical field was assessed using the six-point Fromme-Boezaart scale every five minutes. HR and MAP were recorded throughout. SSC scores were analyzed using the Mann-Whitney U test; continuous variables by Welch’s independent t-test.

Results

Both groups were demographically comparable. SSC scores were significantly lower (better field) in the esmolol group from 5 minutes onward (p < 0.001 at all subsequent time points). Group A reached SSC score 1 (slight bleeding, not a surgical nuisance) by 60 minutes; Group B never reached this level. Surgical duration was shorter in Group A (79.9 ± 15.7 vs. 103.5 ± 15.3 min, p < 0.0001), as was microscope use time (48.2 ± 10.1 vs. 75.7 ± 14.8 min, p < 0.0001). Intraoperative HR was consistently lower in Group A (p < 0.001); MAP was comparable between groups for most of the operative period. No clinically significant adverse events were recorded in either group.

Conclusion

Esmolol is superior to nitroglycerin in achieving a bloodless surgical field in middle ear surgery under general anesthesia. By providing effective HR control alongside adequate MAP reduction, esmolol achieves earlier and more sustained field clarity, significantly shortening surgical and microscope use time without adverse hemodynamic effects.

## Introduction

Tympanoplasty, mastoidectomy, and related middle ear procedures are performed within a confined anatomical space containing critical structures, including the facial nerve, ossicular chain, and the labyrinth of the inner ear. The mandatory use of an operating microscope or endoscope means that even the smallest amount of bleeding is magnified proportionally, rapidly obscuring the operative field. Studies have demonstrated that a reduction in intraoperative bleeding by as little as 28 mL meaningfully improves surgical visibility and surgeon satisfaction [1]. In endoscopic ear surgery, even minor oozing within the confined endoscopic space becomes technically challenging to manage without interrupting the procedure [2].

Controlled hypotension - the deliberate reduction of mean arterial pressure (MAP) to minimize capillary perfusion pressure and thereby reduce intraoperative bleeding - is a well-established anesthetic technique for surgeries requiring a clear field [3]. While MAP reduction is a principal driver of bleeding reduction, emerging evidence highlights that an elevated heart rate (HR) independently increases pulsatile arterial bleeding and counteracts the gains achieved by hypotension alone [4,5]. Ha et al. confirmed that both cardiac output and MAP independently influence surgical field quality during endoscopic procedures [6].

Esmolol, an ultra-short-acting cardioselective beta-1 adrenergic blocker, reduces HR and cardiac output without directly affecting systemic vascular resistance (SVR). Its half-life of approximately nine minutes makes it highly titratable in the perioperative setting [7]. Nitroglycerin (NTG), a nitric oxide donor, acts predominantly on venous capacitance vessels to reduce preload, achieving MAP reduction but frequently inducing compensatory reflex tachycardia through baroreceptor-mediated sympathetic activation, which may partially negate the benefit of reduced pressure [8].

Several randomized controlled trials (RCTs) have compared esmolol and NTG in the context of controlled hypotension for ENT surgeries, particularly functional endoscopic sinus surgery (FESS), with a majority favoring esmolol for field clarity and HR control [9-12]. However, data specific to middle ear surgery are limited. Given the unique hemostatic demands of otological microsurgery, a well-powered RCT with systematic evaluation of both HR and MAP, in conjunction with a validated bleeding scale, is warranted.

This study was designed to compare the efficacy of esmolol and NTG infusions in achieving a bloodless surgical field in middle ear surgery under general anesthesia.

## Materials and methods

Study design and setting

Kalinga Institute of Medical Sciences (KIMS) and Pradyumna Bal Memorial (PBM) Hospital, Kalinga Institute of Industrial Technology (KIIT) University, Bhubaneswar, Odisha, India, over a one-year period. The study was approved by the Institutional Ethics Committee (Ref. No.: KIIT/KIMS/IEC/1580/2024, February 6, 2024) and registered with the Clinical Trials Registry - India (CTRI/2024/04/065218). The study was conducted in accordance with the Declaration of Helsinki and International Council for Harmonisation - Good Clinical Practice (ICH-GCP) guidelines. Written informed consent was obtained from all participants in their preferred language.

Sample size

Sample size was calculated a priori based on the study by Alkan et al. (2021) [13], which reported surgeon satisfaction rates of 60% (esmolol) vs. 30% (NTG). Using a two-proportion z-test (p1=0.60, p2=0.30; alpha=0.05; power=80%), a minimum of 48 patients per group was required, rounded to 50 per group to account for attrition (n=100 total). At the time of protocol registration, no published study had reported the mean ± SD of serial SSC scores for the esmolol vs. NTG comparison in middle ear surgery, precluding a continuous-outcome power calculation; the binary surrogate was therefore the closest available reference. A post-hoc sensitivity analysis using the observed SSC score data confirmed that the study achieved greater than 99% power for the continuous primary endpoint (Cohen's d > 1.0 at all time points from five minutes onward with n=50 per group).

Participants

Inclusion Criteria

Adult patients (≥18 years) of either sex, ASA I or II, scheduled for elective middle ear surgery under general anesthesia. The study included procedure types - myringoplasty, tympanoplasty Type I, tympanoplasty Type II/III, and mastoidectomy.

Exclusion Criteria

Antiplatelet or anticoagulant therapy, bleeding or coagulation disorders, preoperative BP > 140/90 mmHg, resting HR < 60 or > 120 bpm, heart block, reactive airway disease, congestive heart failure, or peripheral vascular disease.

Randomization and blinding

Patients were randomized using a computer-generated sequence and allocated via sequentially numbered, opaque, sealed envelopes. The operating surgeon assessing the surgical field (primary outcome assessor) and all patients were blinded to group allocation throughout. NTG infusions were prepared in non-polyvinyl chloride (PVC) glass bottles with polyethylene administration sets to prevent drug adsorption, while esmolol was delivered via standard PVC tubing. Owing to these physicochemical differences in delivery requirements, complete blinding of the administering anesthesiologist was not achievable; however, the administering anesthesiologist was not involved in SSC scoring or outcome data collection, preserving the integrity of primary outcome assessment.

Interventions

Group A (Esmolol, n=50)

A loading dose of 500 mcg/kg was administered intravenously over one minute, followed by a continuous infusion initiated at 50 mcg/kg/min and titrated up to a maximum of 200 mcg/kg/min to maintain a target MAP of 60-65 mmHg.

Group B (NTG, n=50)

No loading dose was administered. A continuous infusion was initiated at 0.5 mcg/kg/min and titrated up to a maximum of 5 mcg/kg/min to maintain the same MAP target of 60-65 mmHg. NTG infusions were prepared in non-PVC glass bottles with polyethylene administration sets to prevent adsorption.

Anesthetic protocol

Standard preoperative assessment was performed. Monitoring included ECG, SpO_2_, and non-invasive blood pressure.

Premedication

Glycopyrrolate 0.01 mg/kg and nalbuphine 0.1 mg/kg IV. Preoxygenation with 100% O_2_ for three minutes was followed by induction with propofol 2 mg/kg and succinylcholine 1.5 mg/kg.

Maintenance

Isoflurane 0.6-0.8% MAC in N_2_O:O_2_ (2:1). Vecuronium 0.01 mg/kg was given intermittently. IV paracetamol 15 mg/kg and dexamethasone 0.1 mg/kg were administered intraoperatively. Reversal of neuromuscular blockade: neostigmine 0.05 mg/kg with glycopyrrolate 0.01 mg/kg.

Outcome measures

Primary Outcomes

Surgical Site Comfort (SSC) score: Assessed using the six-point Fromme-Boezaart scale (Table 1) [10]. Recorded every five minutes intraoperatively. Analyzed by Mann-Whitney U test (ordinal data; expressed as median with IQR).

**Table 1:** Fromme-Boezaart Surgical Site Comfort (SSC) scoring scale SSC scoring scale adapted from Boezaart et al. (1995) [10].

Score	Description
0	No bleeding – virtually bloodless field
1	Slight bleeding – not a surgical nuisance; no suctioning required
2	Slight bleeding – occasional suctioning required; visibility not impaired
3	Slight bleeding – frequent suctioning required; visibility slightly impaired
4	Moderate bleeding – frequent suctioning required; visibility severely impaired
5	Severe bleeding – constant suctioning required; surgery not possible

Total surgical duration: Time from incision to wound closure (minutes).

Operative microscope use time: Duration of active microscope use (minutes).

Secondary Outcomes

Intraoperative HR: Recorded continuously throughout the procedure. Analyzed by Welch's independent t-test (expressed as mean ± SD).

Intraoperative MAP: Recorded continuously; target range 60-65 mmHg in both groups. Analyzed by Welch's independent t-test.

Preoperative and postoperative hemodynamics: Baseline and recovery-phase HR and MAP, compared between groups.

Adverse events: Clinically significant hemodynamic events, bradycardia, hypotension, or other drug-related complications.

Statistical analysis

Data are presented as mean ± SD for continuous parametric variables and median (IQR) for ordinal variables, and frequency (%) for categorical variables. The normality of continuous data was assessed using the Shapiro-Wilk test prior to analysis; all continuous parametric variables (age, weight, HR, MAP, surgical duration, and microscope use time) conformed to a normal distribution (p > 0.05), justifying the use of parametric tests. SSC scores, being ordinal and non-normally distributed, were analyzed using the Mann-Whitney U test. All continuous parametric variables were compared using Welch’s independent samples t-test, which does not assume equal variances. Categorical variables were compared by Chi-square or Fisher’s exact test. Given the repeated measurements across approximately 14 time points per outcome, Bonferroni correction was applied; the adjusted significance threshold was set at p < 0.0036 (0.05 ÷ 14). All between-group differences for SSC scores and heart rate that were significant at p < 0.05 remained significant after correction (p < 0.001 at every time point). Results for time-point analyses are reported with both uncorrected and Bonferroni-corrected significance noted accordingly; p < 0.05 was considered statistically significant for non-repeated comparisons. All analyses were performed using IBM SPSS Statistics, version 26.0 (IBM Corp., Armonk, NY, USA).

## Results

Baseline characteristics

**Figure 1:**
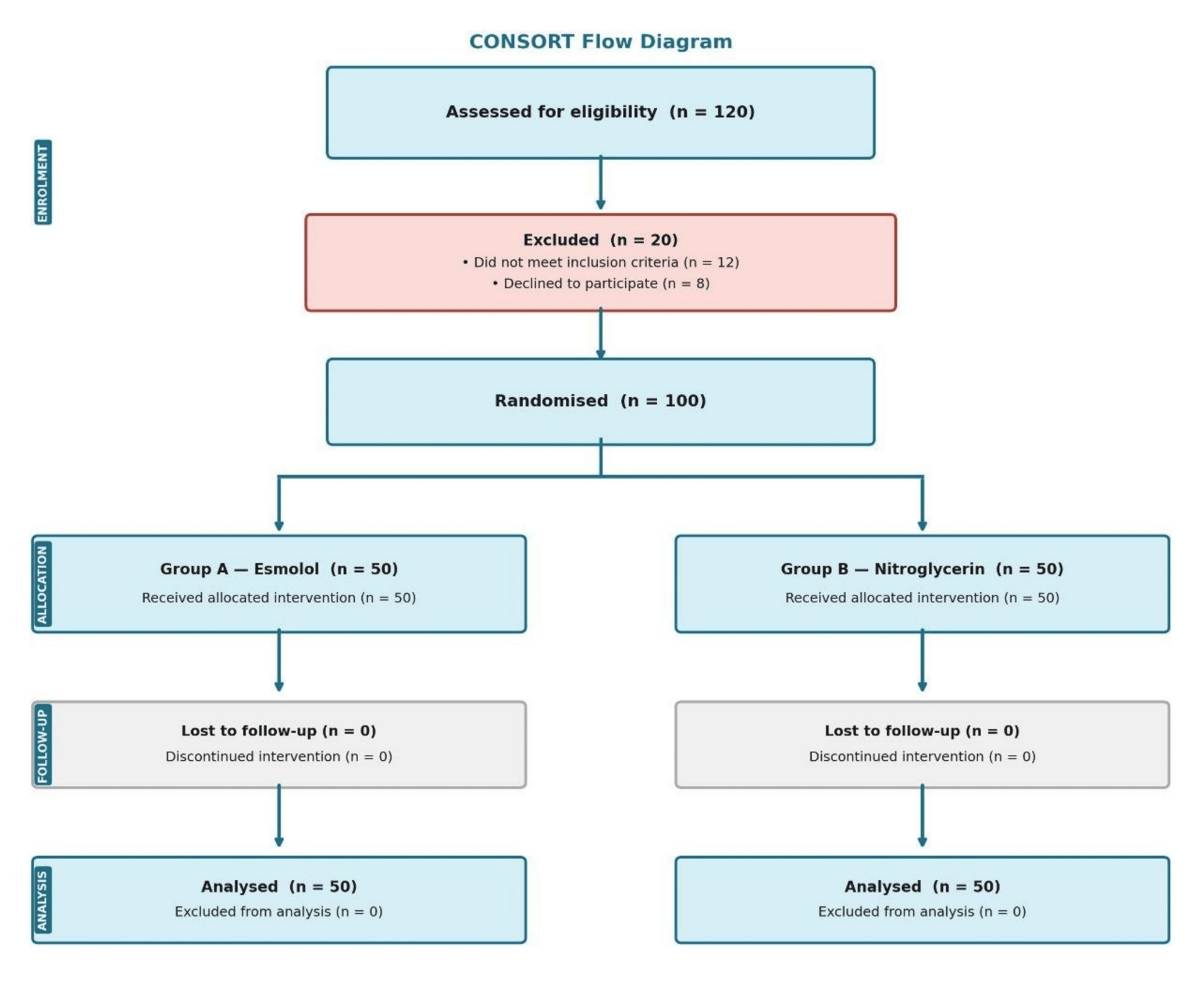
CONSORT flow diagram for RCT reporting; enrollment, allocation, follow-up, analysis CONSORT: Consolidated Standards of Reporting Trials; RCT: randomized controlled trial

One hundred patients were enrolled and equally allocated (Figure 1). Both groups were comparable in baseline demographics. The mean age in Group A was 35.2 ± 12.3 years compared to 35.9 ± 12.7 years in Group B (p = 0.08, NS). Sex distribution was similar, with 27 male and 23 female patients in Group A versus 25 male and 25 female patients in Group B (p = 0.72, NS).

Primary outcomes

Surgical Duration and Microscope Use Time

**Figure 2:**
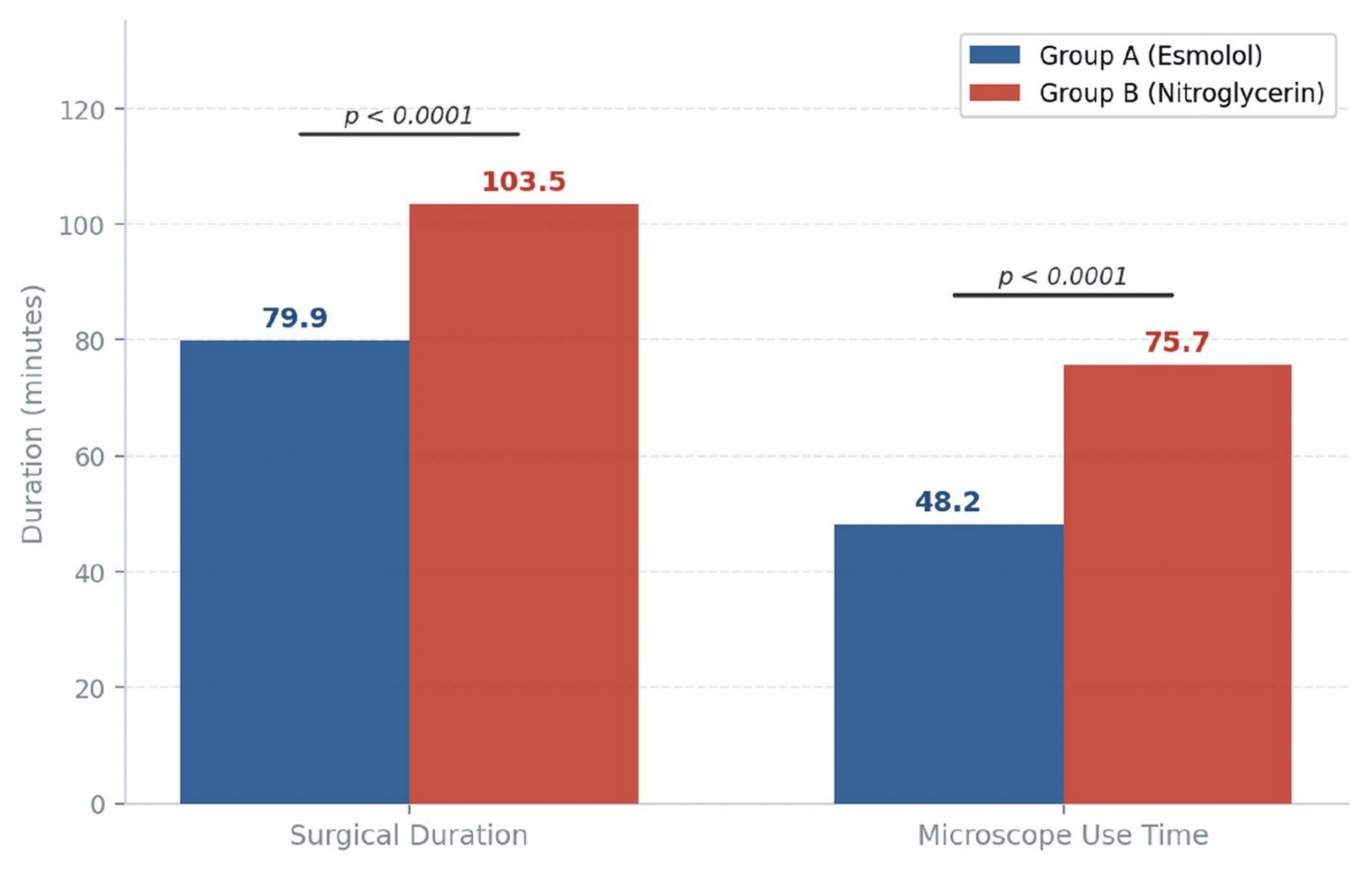
Duration of surgery and microscope use in Group A (esmolol) vs. Group B (nitroglycerin)

As shown in Figure 2, the mean total surgical duration was significantly shorter in Group A (79.9 ± 15.7 vs. 103.5 ± 15.3 min, p < 0.0001). Microscope use time was also significantly reduced (48.2 ± 10.1 vs. 75.7 ± 14.8 min, p < 0.0001), representing a ~57% reduction in operative microscopy time with esmolol.

Surgical Site Comfort Score

**Figure 3:**
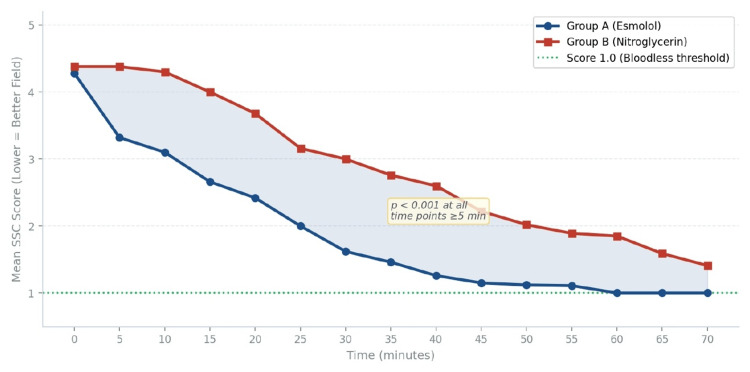
SSC score trajectory over time in Group A (esmolol, blue) vs. Group B (nitroglycerin, red) SSC: Surgical Site Comfort

At drug initiation (0 minutes), SSC scores were comparable between groups (4.28 ± 0.45 vs. 4.38 ± 0.37; p = 0.23). From five minutes onward, scores diverged significantly and remained consistently lower in Group A at every time point (p < 0.001; Table 2, Figure 3). Group A achieved an SSC score of 1 (slight bleeding, not a surgical nuisance) by 60 minutes; Group B never reached this level throughout the observation period.

**Table 2:** : Comparative assessment of SSC score over time SSC: Surgical Site Comfort; NS: non-significant * Statistically significant (p < 0.001)

Time (min)	Group A Mean ± SD	Group B Mean ± SD	U-value	P value	Sig.
0	4.28 ± 0.45	4.38 ± 0.37	U = 1424	0.23	NS
5	3.32 ± 0.59	4.38 ± 0.53	U > 1727	< 0.001	*
10	3.10 ± 0.65	4.30 ± 0.51	U > 1727	< 0.001	*
15	2.66 ± 0.66	4.00 ± 0.64	U > 1727	< 0.001	*
20	2.42 ± 0.64	3.68 ± 0.59	U > 1727	< 0.001	*
30	1.62 ± 0.67	3.00 ± 0.64	U > 1727	< 0.001	*
40	1.26 ± 0.47	2.60 ± 0.61	U > 1727	< 0.001	*
50	1.12 ± 0.27	2.02 ± 0.43	U > 1727	< 0.001	*
60	1.00 ± 0.00	1.85 ± 0.45	U > 1727	< 0.001	*
70	1.00 ± 0.00	1.41 ± 0.43	U > 1727	< 0.001	*

Secondary outcomes

Preoperative and Postoperative Hemodynamics

Preoperative HR and MAP did not differ significantly between groups, confirming baseline comparability. Postoperative hemodynamics were also comparable, indicating stable recovery in both groups (Table 3). The wider postoperative MAP variability in Group B (SD 17.2 vs. 9.1 mmHg) is consistent with NTG’s pharmacodynamic profile; abrupt cessation of NTG infusion may trigger rebound sympathetic activation and residual vasodilatory instability, contributing to greater MAP fluctuation in the early postoperative period.

**Table 3:** Preoperative and postoperative HR and MAP HR: heart rate; MAP: mean arterial pressure; NS: non-significant

Parameter	Group A Mean ± SD	Group B Mean ± SD	Test statistic	P value	Sig.
Pre-op HR (bpm)	82.6 ± 13.0	87.0 ± 10.6	t = −1.85	0.29	NS
Post-op HR (bpm)	80.9 ± 10.9	86.6 ± 11.8	t = −2.51	0.767	NS
Pre-op MAP (mmHg)	85.4 ± 7.8	84.4 ± 8.9	t = 0.60	0.567	NS
Post-op MAP (mmHg)	81.4 ± 9.1	83.5 ± 17.2	t = −0.76	0.453	NS

Intraoperative Heart Rate

**Figure 4:**
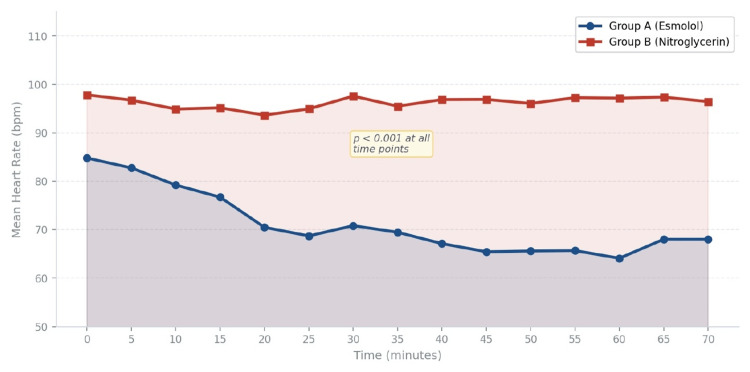
Intraoperative heart rate over time

Group A maintained significantly lower HR throughout the operative period (Table 4, Figure 4). At drug initiation, HR was 84.78 ± 9.72 bpm in Group A vs. 97.82 ± 10.20 bpm in Group B (p < 0.001). This differential was sustained throughout, with Group A stabilizing at approximately 65-70 bpm while Group B maintained 95-98 bpm. All time-point differences were statistically significant (p < 0.001).

**Table 4:** Comparative assessment of intraoperative HR HR: heart rate

Time (min)	Group A HR Mean ± SD (bpm)	Group B HR Mean ± SD (bpm)	t-value	P value
0	84.78 ± 9.72	97.82 ± 10.20	−6.54	< 0.001
10	79.20 ± 7.17	94.88 ± 6.29	−11.62	< 0.001
20	70.48 ± 8.57	93.64 ± 5.92	−15.72	< 0.001
30	70.82 ± 11.32	97.56 ± 6.84	−14.30	< 0.001
40	67.11 ± 5.10	96.84 ± 4.85	−29.87	< 0.001
50	65.58 ± 3.38	96.04 ± 6.02	−31.20	< 0.001
60	64.09 ± 2.34	97.14 ± 5.87	−36.98	< 0.001
70	68.00 ± 0.20	96.43 ± 4.34	−46.27	< 0.001

Intraoperative Mean Arterial Pressure

**Figure 5:**
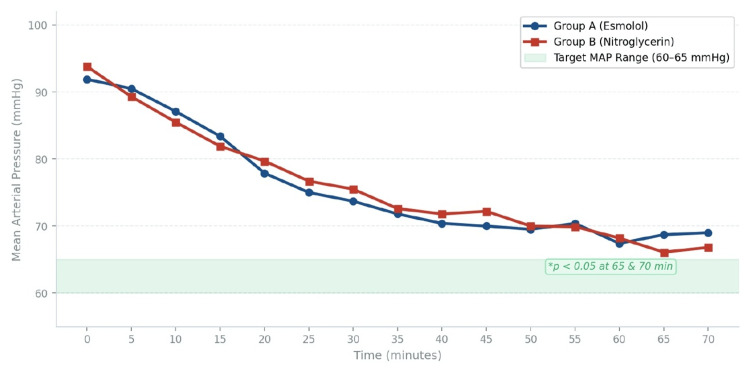
: Intraoperative MAP over time

Both groups achieved comparable MAP reductions with no statistically significant intergroup differences from 0-60 minutes (Table 5, Figure 5). Both groups declined from baseline (~91-94 mmHg) to the targeted range (~67-70 mmHg) by 60 minutes. Statistically significant differences appeared only at 65 minutes (p = 0.011) and 70 minutes (p = 0.030), where MAP was marginally lower in Group B, likely reflecting persistent NTG vasodilation at late time points.

**Table 5:** Comparative assessment of intraoperative MAP MAP: mean arterial pressure; NS: non-significant * Statistically significant (p < 0.001)

Time (min)	Group A MAP Mean ± SD (mmHg)	Group B MAP Mean ± SD (mmHg)	t-value	P value
0	91.9 ± 7.0	93.8 ± 8.1	−1.25	0.212 (NS)
10	87.1 ± 8.9	85.5 ± 10.5	0.82	0.389 (NS)
20	77.9 ± 8.5	79.7 ± 8.8	−1.04	0.284 (NS)
30	73.7 ± 7.7	75.5 ± 7.9	−1.15	0.238 (NS)
40	70.4 ± 7.1	71.8 ± 6.9	−1.00	0.338 (NS)
50	69.5 ± 5.4	70.0 ± 6.8	−0.41	0.684 (NS)
60	67.4 ± 8.6	68.2 ± 2.0	−0.64	0.603 (NS)
65	68.7 ± 6.9	66.1 ± 3.2	2.42	0.011*
70	69.0 ± 6.8	66.8 ± 1.7	2.22	0.030*

Adverse Events

No clinically significant adverse events were recorded in either group throughout the study period (Table 6). No patient in Group A required atropine for symptomatic bradycardia. No patient in Group B required vasopressor support for refractory hypotension. No episodes of bronchospasm or drug hypersensitivity were observed in either group.

**Table 6:** Adverse events HR: heart rate; MAP: mean arterial pressure; NTG: nitroglycerin

Adverse events	Group A (esmolol) n=50	Group B (NTG) n=50	P value
Bradycardia (HR < 50 bpm) requiring atropine	0	0	NS
Severe hypotension (MAP < 55 mmHg) requiring vasopressor	0	0	NS
Rebound tachycardia (HR > 110 bpm post-infusion)	0	0	NS
Bronchospasm	0	0	NS
Drug hypersensitivity/anaphylaxis	0	0	NS

## Discussion

This randomized controlled trial provides prospective, double-blinded evidence demonstrating the superiority of esmolol over nitroglycerin in achieving a high-quality bloodless surgical field in middle ear surgery. Esmolol’s advantage was evident from as early as five minutes after drug initiation, with SSC scores significantly and consistently lower throughout the operative period - a finding with direct clinical relevance to surgical precision in the confined middle ear space [14].

The critical importance of a dry surgical field in otological microsurgery cannot be overstated. Even a 28 mL reduction in blood loss has been shown to significantly improve surgeon satisfaction and field visibility in meta-analytic data. Under operating microscope magnification, the effective visual impact of any blood is amplified manifold, making the benefit of a drier field even more pronounced than absolute volume measurements would suggest [15].

The principal mechanism underlying esmolol’s advantage over NTG appears to be its cardiac chronotropic action. While both agents achieved equivalent MAP reductions for most of the operative period, esmolol maintained a consistently lower HR (~65-70 bpm vs. ~95-98 bpm, p < 0.001 at all time points). A sustained elevated HR in the NTG group - despite comparable arterial pressure - results in greater cardiac output and pulsatile capillary flow, partially negating the hemostatic benefit of pressure reduction. This mechanistic difference, rather than any superior ability to lower MAP, accounts for esmolol’s advantage.

These findings align with earlier trials. Boezaart et al. demonstrated that esmolol achieved ideal operative field scores at a MAP above 65 mmHg during FESS, while sodium nitroprusside required deeper hypotension to achieve inferior scores [10]. Srivastava et al. confirmed in a FESS trial that esmolol achieved ideal conditions at a higher MAP with lower HR and blood loss [9]. Gowtham et al. specifically examined reported shorter surgical duration and improved field visibility with esmolol versus NTG, directly corroborating the present study’s findings [8].

The reduction in total surgical duration (79.9 vs. 103.5 minutes) and microscope use time (48.2 vs. 75.7 minutes) in our study is larger in absolute magnitude than the reductions reported by Gowtham et al. and Srivastava et al. [8,9]. We attribute this to several factors: (1) middle ear microsurgery imposes uniquely stringent haemostatic requirements compared to FESS - the confined bony operative space does not permit repeated wide-field suction, and a single bleeding episode can necessitate prolonged stoppage and field clearing; (2) our primary comparison was microscope use time specifically, which directly amplifies the effect of field quality differences; and (3) baseline SSC scores in our cohort (4.28-4.38 at initiation) were relatively high, suggesting a greater absolute margin for improvement with an effective agent.

The MAP data further support the clinical interpretation. Despite esmolol reducing MAP through a cardiac output-mediated mechanism rather than peripheral vasodilation, both agents achieved comparable target MAP reductions. Esmolol’s advantage is thus entirely attributable to the additive benefit of HR reduction on field quality, independent of MAP [9].

The wider postoperative MAP variability in the NTG group (SD 17.2 vs. 9.1 mmHg) is consistent with NTG’s pharmacodynamic profile. Abrupt cessation of nitroglycerin can trigger rebound sympathetic activation and residual vasodilatory instability, contributing to MAP fluctuation in early recovery. Esmolol’s withdrawal is associated with gradual HR normalization without a pronounced rebound effect, explaining the more stable postoperative MAP trajectory in Group A [16-18].

From a safety perspective, pre- and postoperative hemodynamic parameters were comparable between groups, and no clinically significant adverse events were recorded in either group (Table 3), consistent with the known safety profile of titrated esmolol infusion in ASA I-II patients [19].

Limitations of this study include a single-center design and restriction to ASA I-II adults. Second, the SSC assessment involved multiple operating surgeons over the study period; formal inter-observer reliability testing (e.g., intraclass correlation coefficient) was not performed, and inter-observer variability may have introduced assessment bias. Third, complete blinding of the administering anesthesiologist was not feasible due to the differing infusion delivery requirements of the study drugs (glass/polyethylene for NTG vs. standard PVC tubing for esmolol); the primary outcome assessor (operating surgeon) remained blinded throughout. Fourth, the sample size calculation was based on a binary surrogate endpoint; although post-hoc analysis confirms adequate power for the continuous SSC outcome, prospective calculation using SSC mean ± SD would strengthen future trials. Fifth, quantitative blood loss measurement was not performed; future studies with standardized video-assisted field quantification and actual blood loss volumes would further strengthen the evidence base. Sixth, the study was restricted to ASA I-II adults; findings may not be directly applicable to higher-risk patients or pediatric populations.

## Conclusions

Esmolol is superior to nitroglycerin in providing a bloodless surgical field in middle ear surgery under general anesthesia. By combining effective heart rate control with adequate MAP reduction, esmolol achieves earlier, more complete, and more sustained improvements in surgical field quality as assessed by the Fromme-Boezaart scale. This translates into significantly shorter total surgical duration and reduced operative microscope use time, with a comparable and stable hemodynamic profile. These findings support the preferential use of esmolol for controlled hypotension in otological microsurgery.
